# COVID-19 infection or vaccination and hidradenitis suppurativa: A systematic review

**DOI:** 10.1016/j.jdin.2024.09.004

**Published:** 2024-10-11

**Authors:** Alim Osman, Megan Jayne Ralston, Michael Christopher Povelaitis, Mariela Mitre

**Affiliations:** a School of Medicine, Eastern Virginia Medical School, Norfolk, Virginia; b Hackensack Meridian School of Medicine, Nutley, New Jersey; c Division of Dermatology, Department of Medicine, Hackensack University Medical Center, Hackensack, Nutley

**Keywords:** autoimmune flares, biologic, COVID-19, dermatology, hidradenitis suppurativa, HS

*To the Editor:* Hidradenitis suppurativa (HS) is a chronic inflammatory skin condition with a complex treatment regimen.^1^ COVID-19 posed significant challenges for patients with chronic conditions, with recent studies examining its effect on HS.^2^ Most studies did not find significant differences between COVID-infected HS patients and controls. However, some evidence suggests increased HS exacerbations after receiving the COVID vaccine. This highlights the importance of personalized care strategies for HS patients and the need for further research on the relationship between COVID and HS.

This study was conducted in accordance with the Preferred Reporting Items for Systematic Reviews and Meta-Analyses (PRISMA) (Fig 1). Our complete methodology with inclusion/exclusion criteria on Mendeley (https://data.mendeley.com/datasets/yrg5v7pcck/2). Of the 9 studies included, most were retrospective in nature (Supplementary Table I, available via Mendeley at https://data.mendeley.com/datasets/yrg5v7pcck/2). The majority of studies did not find significant differences in death or hospitalization rates between COVID-infected HS patients and controls. A complete summary of each study with significant findings is found in Supplementary Table II, available via Mendeley at https://data.mendeley.com/datasets/yrg5v7pcck/2, with citations of each included study also available on Mendeley (https://data.mendeley.com/datasets/yrg5v7pcck/2).

**Fig 1:**
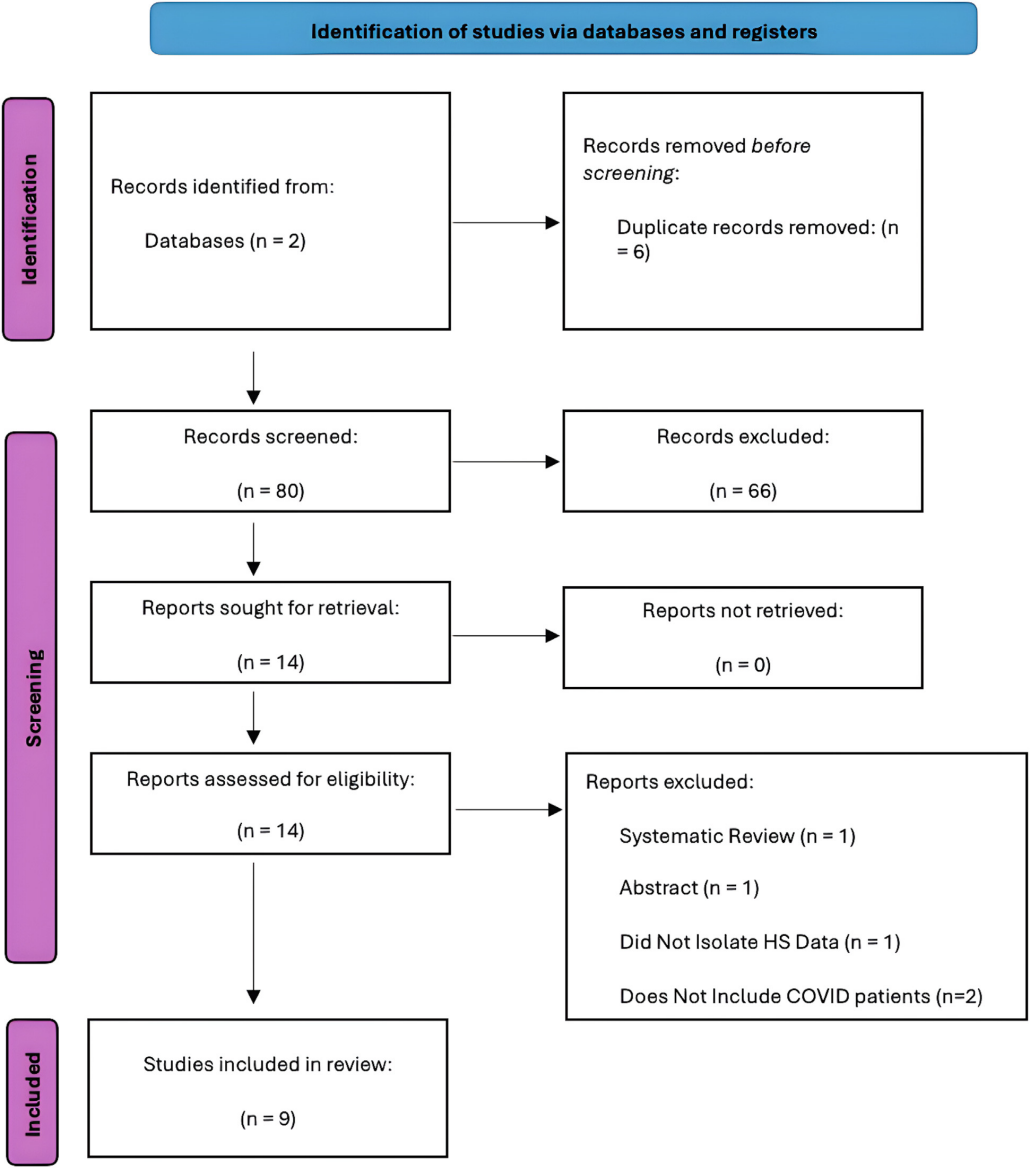
PRISMA flow chart of study inclusion. *PRISMA*, Preferred Reporting Items for Systematic Reviews and Meta-Analyses; *HS*, hidradenitis suppurativa.

However, of the 2 vaccine studies, both found increased HS exacerbations following vaccine administration. Liakou et al assessed exacerbations using several scoring systems including the International Hidradenitis Suppurativa Severity Score System and the Dermatology Quality of Life Index.^3^ HS flares were identified as new or significantly worsened clinical signs or symptoms occurring within 30 days after vaccination.^3^ They observed that 48 out of 250 (19.2%) HS patients who received a COVID vaccine experienced exacerbations, with a particular increase among those who received mRNA vaccines.^3^ This finding was corroborated by Martora et al, who recorded an increase in HS symptoms and severity on the International Hidradenitis Suppurativa Severity Score System scoring in 5 cases following each vaccine dose.^4^ Given that HS is an autoinflammatory condition, these findings suggest that the COVID vaccine, particularly the mRNA vaccine, may activate pro-inflammatory pathways that contribute to flare-ups in chronic inflammatory diseases like HS.^4^ However, there appears to be a higher risk of HS symptom exacerbations associated with mRNA COVID-19 vaccines. This could be due to several factors, including molecular mimicry and the immune system's response to the vaccine.^5^ Further research is needed to understand the mechanisms behind these exacerbations and to develop optimal treatment protocols for HS patients.

Due to data heterogeneity, only qualitative analysis was possible, limiting the strength of the conclusions. Nonetheless, the review highlights the need for personalized care strategies for HS patients during the COVID-19 pandemic and throughout future vaccination cycles. Further studies are needed to evaluate the relationship between COVID-19 vaccines and HS outcomes to optimize treatment protocols and enhance patient care.

Future research should continue to explore the relationship between COVID-19 vaccines and HS to provide clearer guidance for managing HS patients during vaccination campaigns. Limitations include keywords used for article identification. Different keywords/databases may yield different results. Given the waxing and waning nature of HS, exacerbations following vaccination may not be attributable to the vaccination itself.

## Conflicts of interest

None disclosed.
